# Exploratory Analysis of Serial ^18^F-fluciclovine PET-CT and Multiparametric MRI during Chemoradiation for Glioblastoma

**DOI:** 10.3390/cancers14143485

**Published:** 2022-07-18

**Authors:** Kavi Fatania, Russell Frood, Marcus Tyyger, Garry McDermott, Sharon Fernandez, Gary C. Shaw, Marjorie Boissinot, Daniela Salvatore, Luisa Ottobrini, Irvin Teh, John Wright, Marc A. Bailey, Joanna Koch-Paszkowski, Jurgen E. Schneider, David L. Buckley, Louise Murray, Andrew Scarsbrook, Susan C. Short, Stuart Currie

**Affiliations:** 1 Department of Radiology, Leeds Teaching Hospitals Trust, Leeds General Infirmary, Leeds LS1 3EX, UK; russellfrood@nhs.net (R.F.); a.scarsbrook@nhs.net (A.S.); stuartcurrie@nhs.net (S.C.); 2 Leeds Institute of Medical Research, University of Leeds, Leeds LS2 9TJ, UK; gary_shaw12@hotmail.com (G.C.S.); M.Boissinot@leeds.ac.uk (M.B.); louise.murray8@nhs.net (L.M.); susan.short4@nhs.net (S.C.S.); 3 Department of Medical Physics, Leeds Teaching Hospitals Trust, St James’s University Hospital, Leeds LS9 7TF, UK; marcus.tyyger@nhs.net (M.T.); garry.mcdermott@nhs.net (G.M.); 4 Department of Clinical Oncology, Leeds Teaching Hospitals Trust, St James’s University Hospital, Leeds LS9 7TF, UK; sharon.fernandez1@nhs.net; 5 Department of Pathophysiology and Transplantation, University of Milan, 20122 Segrate, Italy; Daniela.salvatore@unimi.it (D.S.); luisa.ottobrini@unimi.it (L.O.); 6 Institute of Molecular Bioimaging and Physiology, National Research Council (IBFM-CNR), 20054 Segrate, Italy; 7 Biomedical Imaging Science Department, and Discovery & Translational Science Department, Leeds Institute of Cardiovascular and Metabolic Medicine, University of Leeds, Leeds LS2 9TJ, UK; I.Teh@leeds.ac.uk (I.T.); J.Wright2@hull.ac.uk (J.W.); M.A.Bailey@leeds.ac.uk (M.A.B.); J.Koch-Paszkowski@leeds.ac.uk (J.K.-P.); J.E.Schneider@leeds.ac.uk (J.E.S.); D.L.Buckley@leeds.ac.uk (D.L.B.); 8 Leeds Vascular Institute, Leeds Teaching Hospitals Trust, Leeds General Infirmary, Leeds LS1 3EX, UK

**Keywords:** glioblastoma, chemoradiotherapy, adjuvant, positron-emission tomography, magnetic resonance imaging, amino acid transport systems

## Abstract

**Simple Summary:**

Glioblastoma (GBM), the most common malignant adult primary brain tumour, has a prognosis of ~12–15 months. Poor prognosis is partly due to the inability to accurately define the extent of tumour infiltration; currently demarcated using magnetic resonance imaging (MRI) sequences (e.g., post-contrast T1-weighted (Gd-T1) and dynamic contrast-enhanced (DCE-MRI)). Anti-1-amino-3-^18^fluorine-fluorocyclobutane-1-carboxylic acid (^18^F-fluciclovine) positron emission tomography (PET) may depict GBM better than MRI. This prospective pilot study aimed to explore the relationship of ^18^F-fluciclovine PET, DCE-MRI and Gd-T1 in patients with GBM undergoing standard-of-care adjuvant chemoradiotherapy. A parallel mouse glioma model was used to investigate the relationship between ^18^F-fluciclovine PET, MRI and tumour biology. Clinical results showed that GBM volume on ^18^F-fluciclovine PET tended to be larger than Gd-T1 and DCE-MRI in patients with shorter overall survival (OS) but smaller in patients with longer OS. The preclinical study confirmed that ^18^F-fluciclovine uptake reflected biologically active tumour. Results suggest that ^18^F-fluciclovine PET may better define GBM infiltration than MRI.

**Abstract:**

Anti-1-amino-3-^18^fluorine-fluorocyclobutane-1-carboxylic acid (^18^F-fluciclovine) positron emission tomography (PET) shows preferential glioma uptake but there is little data on how uptake correlates with post-contrast T1-weighted (Gd-T1) and dynamic contrast-enhanced magnetic resonance imaging (DCE-MRI) activity during adjuvant treatment. This pilot study aimed to compare ^18^F-fluciclovine PET, DCE-MRI and Gd-T1 in patients undergoing chemoradiotherapy for glioblastoma (GBM), and in a parallel pre-clinical GBM model, to investigate correlation between ^18^F-fluciclovine uptake, MRI findings, and tumour biology. ^18^F-fluciclovine-PET-computed tomography (PET-CT) and MRI including DCE-MRI were acquired before, during and after adjuvant chemoradiotherapy (60 Gy in 30 fractions with temozolomide) in GBM patients. MRI volumes were manually contoured; PET volumes were defined using semi-automatic thresholding. The similarity of the PET and DCE-MRI volumes outside the Gd-T1 volume boundary was measured using the Dice similarity coefficient (DSC). CT-2A tumour-bearing mice underwent MRI and ^18^F-fluciclovine PET-CT. Post-mortem mice brains underwent immunohistochemistry staining for ASCT2 (amino acid transporter), nestin (stemness) and Ki-67 (proliferation) to assess for biologically active tumour. 6 patients were recruited (GBM 1–6) and grouped according to overall survival (OS)—short survival (GBM-SS, median OS 249 days) and long survival (GBM-LS, median 903 days). For GBM-SS, PET tumour volumes were greater than DCE-MRI, in turn greater than Gd-T1. For GBM-LS, Gd-T1 and DCE-MRI were greater than PET. Tumour-specific ^18^F-fluciclovine uptake on pre-clinical PET-CT corresponded to immunostaining for Ki-67, nestin and ASCT2. Results suggest volumes of ^18^F-fluciclovine-PET activity beyond that depicted by DCE-MRI and Gd-T1 are associated with poorer prognosis in patients undergoing chemoradiotherapy for GBM. The pre-clinical model confirmed ^18^F-fluciclovine uptake reflected biologically active tumour.

## 1. Introduction

Defining the tumour edge in glioma is an essential component of target volume delineation for radiotherapy. In conventional practice, gadolinium-enhancement on T1-weighted magnetic resonance (MR) sequences (Gd-T1), which represents areas of blood brain barrier (BBB) breakdown, together with the post-operative tumour cavity are used to define the macroscopic tumour edge. It is accepted, however, that this approach is insufficient to determine the true extent of infiltrative tumour cells [1,2]. As such, uniform margins are added to the delineated macroscopic tumour to take account of potential microscopic spread. Such margins inevitably result in irradiation of the normal brain and as such limit the radiotherapy dose that can be delivered. Better understanding of the glioma tumour edge could facilitate a more personalised approach to radiotherapy treatment planning, potentially enhancing the therapeutic ratio.

Positron emission tomography (PET) using anti-1-amino-3-^18^fluorine-fluorocyclobutane-1-carboxylic acid (^18^F-fluciclovine), a synthetic L-leucine analogue shows preferential glioma cell uptake with low activity in the normal brain [3,4,5]. ^18^F-fluciclovine is transported into glial cells by L-amino acid transporters and alanine-serine-cysteine transporters that are upregulated in neoplastic glial cells [6,7]. Studies have demonstrated ^18^F-fluciclovine uptake within glioma tissue that is not Gd-T1 hyperintense and suggests that its uptake may not be an epiphenomenon of a breakdown in the BBB [4,8,9]. The results from multicentre phase 3 trials in glioma patients undergoing both pre-operative MRI and ^18^F-fluciclovine PET-computed tomography (PET-CT) have demonstrated the clinical utility of ^18^F-fluciclovine, leading to changes in surgical resection extent in over 40% of patients [10]. Animal models of diffuse glioma have also reported that ^18^F-fluciclovine uptake is not dependent upon areas of BBB breakdown and may be correlated with glioma cell density [11].

Dynamic contrast-enhanced perfusion-weighted imaging (DCE) MRI has also been used to investigate vascular changes in glioblastoma (GBM), and studies have shown a correlation with tumour infiltrating beyond the enhancing margin and with markers of tissue hypoxia and neoangiogenesis [2,12,13]. In addition, some DCE-MRI quantitative biomarkers such as interstitial volume (v_e_) may correlate with overall survival (OS) and tumour-associated microvascular proliferation [12]. Past studies of DCE-MRI during adjuvant chemoradiotherapy treatment have examined changes in DCE-MRI parameters such as transfer coefficient (K^trans^), and the relationship to dynamic susceptibility contrast MRI (DSC-MRI) or to diffusion-weighted (DW) MRI [14,15,16]. However, the relationship between DCE-MRI, ^18^F-fluciclovine and Gd-T1 uptake during adjuvant chemoradiotherapy has not been explored. Additionally, there is a lack of published data on how ^18^F-fluciclovine uptake changes with chemoradiotherapy and whether any alteration in uptake correlates with changes in Gd-T1 and DCE-MRI activity.

The aim of this pilot study was to assess concordance between ^18^F-fluciclovine activity and MRI parameters (Gd-T1 and DCE-MRI) in patients with GBM over the course of chemoradiotherapy. In particular, the aim was to assess whether areas of ^18^F-fluciclovine uptake exist outside BBB breakdown (as marked by Gd-T1 hyperintensity) before, during and after adjuvant chemoradiotherapy for patients with GBM. A secondary aim was to examine whether any such areas of ^18^F-fluciclovine uptake outside of BBB breakdown correlate with areas of DCE-MRI uptake. In addition, correlation of tumour uptake of ^18^F-fluciclovine with relevant glioma cell biology in a pre-clinical glioma model was assessed.

## 2. Materials and Methods

### 2.1. Patient Selection and Assessment

Adult patients referred to a tertiary neurosciences centre, which has a catchment area of 3.9–4.4 million adults, with histologically proven World Health Organization (WHO) grade 4 glioblastoma diagnosed between June 2018 and May 2019 and who were suitable for adjuvant chemoradiotherapy (CRT), 60 Gy in 30 fractions with concurrent temozolomide, following surgery or biopsy, were eligible. The study was approved by the Research Ethics Committee (reference 17/YH/0372) and all patients gave written informed consent. The study protocol was registered on Clinicaltrials.gov (ID RD17/101925, Clinicaltrials.gov identifier: NCT03409549). The recruitment target was 12 patients, however, due to the unexpected cessation of ^18^F-fluciclovine radiopharmaceutical supply in July 2019 and the impact of the subsequent COVID-19 pandemic only 6 patients were enrolled. All treatment and clinical assessments were as per standard of care. Overall survival (OS) was calculated from date of surgery to the date of death or to the censor date (14 June 2021).

### 2.2. Clinical Imaging

#### 2.2.1. Imaging Timeline

Patients underwent multiparametric MRI (mpMRI) and ^18^F-fluciclovine PET-CT at three timepoints: (1) Following surgery and prior to CRT (pre-RT), (2) during week 3 of CRT (after 10 fractions; mid-RT), and (3) 6-weeks following completion of CRT (post-RT). PET-CT was acquired within 21 days of the mpMRI study.

#### 2.2.2. MRI Acquisition

All examinations were performed on a 3 Tesla Siemens Prisma MRI scanner (Siemens, Erlangen, Germany). Patients were scanned whilst wearing a radiotherapy mask to mimic treatment conditions and therefore spine and flexible body coils were used for signal reception. The MRI protocol for structural sequences included: axial 3D T2-weighted fluid attenuation inversion recovery (FLAIR) sequence (TR/TE/TI 5000/387/1800 ms; 230 × 230 mm FOV, 1 mm slice thickness; 256 × 256 matrix), 2D axial T1-weighted spin echo sequence pre- and post-contrast using a gadolinium-based contrast agent (DOTAREM^R^, gadoterate meglumine, Guerbet, Gorinchem, The Netherlands) (TR/TE 600/6 ms; 256 × 224 mm FOV, 4 mm slice thickness; 256 × 168 matrix), 3D axial T1-weighted magnetization-prepared rapid gradient echo (MP-RAGE) sequence post-contrast (TR/TE/TI 1900/2.28/1100 ms; 256 × 224 mm field of view (FOV), 1.1 mm slice thickness; 224 × 224 matrix, flip angle 15 degrees). DCE-MRI acquisition consisted of a 3D T1-weighted gradient echo series during injection of 0.1 mmol/kg of Dotarem (TR/TE 3.62/1.24 ms; 256 × 224 mm FOV, slice thickness 4 mm; 128 × 84 matrix; flip angle 25 degrees, temporal resolution 3 s, overall acquisition time 4 min). Inversion recovery TurboFLASH images were acquired pre-contrast with inversion times 83, 400, 1500 and 2890 ms for T1 mapping. For each study timepoint, a neuroradiologist (with >10 years’ experience) reviewed the images in conjunction with the clinical details and prior imaging to provide a report and their impression of whether the imaging showed stability, response to treatment or disease progression. During this interpretation, the neuroradiologist did not refer to DCE-MRI sequences, which were processed later as described below.

#### 2.2.3. PET-CT Acquisition

**Figure 1:**
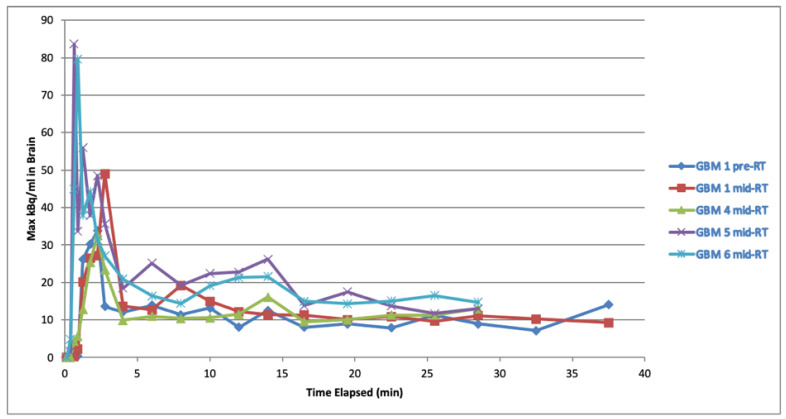
Time-activity curves for whole brain activity following ^18^F-fluciclovine injection.

PET-CT imaging of the head was performed on a GE Healthcare Discovery 690 PET-CT scanner (GE Healthcare, Chicago, IL, USA). Anti-1-amino-3-^18^fluorine-fluorocyclobutane-1-carboxylic acid (^18^F-fluciclovine, Blue Earth Diagnostics Ltd., Oxford, UK) was administered intravenously at a dose of 185 MBq ± 20% and flushed through with saline. The CT component was acquired with the following settings and was also used for attenuation correction: 120 kV; 140 mAs; tube rotation time 0.5 s per rotation; pitch 0.531:1 (10.62/rotation); slice thickness 3.75 mm; 50 cm displayed FOV; 512 × 512 matrix. Following ^18^F-fluciclovine injection, for a subset of patients, a continuous 30-min dynamic PET acquisition was performed in list mode acquisition (frames 4 × 15 s, 4 × 30 s, 6 × 2 min, 5 × 3 min). Dynamic PET reconstruction parameters were: 50 cm displayed FOV, Vue Point HD reconstruction algorithm (GE Healthcare), 24 subsets, 2 iterations, 3.2 mm cut-off filter, 128 × 128 matrix. The patients fasted for at least 4-h before administration of ^18^F-fluciclovine. Time-activity curves (maximum kBq/mL in the brain vs. time) demonstrated a stable equilibrium of activity after 5 min (Figure 1).

A 10-min static image was reconstructed based upon a summed 5–15 min window using 50 cm displayed FOV, time-of-flight Vue Point FX reconstruction algorithm (GE Healthcare), 24 subsets, 2 iterations, 3.2 mm cut-off filter, 128 × 128 matrix. The PET-CT imaging was reviewed by a dual-certified radiologist and nuclear medicine physician (>15 years’ experience) who provided a report and impression of whether the imaging was stable and showed response to treatment or disease progression.

#### 2.2.4. Image Post-Processing and Analysis

DCE-MRI was processed using specialised software, PMI (Platform for Research in Medical Imaging, version 0.4) to generate perfusion-related maps based on the DCE-MRI series and an arterial input function (AIF), delineated from the anterior cerebral artery. Maps of interstitial volume per unit volume of tissue (v_e_) and volume transfer constant between blood plasma and extracellular extravascular space (K^trans^) were generated using the Tofts model, per patient per timepoint [17]. DCE-MRI parameter maps were exported as JPEG images from PMI, cropped to remove the colour scale and converted to Digital Imaging and Communications in Medicine (DICOM) format using the pydicom (v. 2.1.2) [18], numpy (v. 1.21.0) [19] and scikit-image (v. 0.18.0) [20] packages.

Tumour regions of interest (ROI) were segmented using Mirada RTx Advanced (v1.8.2.34893, Mirada Medical Ltd., Oxford, UK). The MRI sequences including v_e_ and K^trans^ parameter maps, and 3D Gd-T1 sequence were manually segmented once by one of two radiology trainees (each with 4 years of radiology experience) and, to limit inter-observer variability, each volume was checked by a single neuroradiologist (>10 years’ experience). The minimum volumes thresholds on Mirada RTx Advanced are limited by the voxel size for each modality, which are 1.26 mm^3^ for Gd-T1, 21.3 mm^3^ for DCE-MRI, and 48.75 mm^3^ for PET.

PET images were contoured using a semi-automatic method also within Mirada RTx Advanced. An 11 cm^3^ region of interest was centred on contralateral normal brain tissue, ensuring it included both grey and white matter, and the maximum standardised uptake value (SUV_max_) of the background was determined. Three different PET tumour volumes were then delineated by setting thresholds at 2, 3 and 4 times the SUV_max_ of background tissue (2 × SUV_max_, 3 × SUV_max_ and 4 × SUV_max_).

#### 2.2.5. Tumour Volume Analysis

For each patient and timepoint, the v_e_, K^trans^ and PET-CT images were all rigidly aligned to the 3D Gd-T1 sequence using RayStation (8B DTK, RaySearch Laboratories, Stockholm, Sweden). The quality of registration was assessed by a Clinical Scientist specialised in radiotherapy planning (with 3 years of experience). The absolute volume of each delineation was determined (cm^3^). Group-wise differences in the volumes across the modalities were tested for statistical significance using the Kruskal–Wallis test using the scipy (v1.7.3) package [21]. Next, the Gd-T1 volume was subtracted from the DCE-MRI and PET volumes to highlight areas of uptake outside of the enhancing tumour margin and thus, outside of BBB breakdown. Subtracted volumes were compared with one another using Dice similarity coefficient (DSC) using RayStation. DSC values range from 0, indicating no spatial overlap between subtracted volumes to 1, indicating complete overlap.

### 2.3. Pre-Clinical Glioma Model

Animal experiments were performed in compliance with the Animals (Scientific Procedures) Act 1986, under UK Home Office license number PA67C4EBE4 which was also approved by the Animal Welfare and Research Ethics Board at the University of Leeds. All animals were housed in Tecniplast green line caging in a pathogen-free environment, given 3R’s bedding made from sterile recycled paper material and fed a diet of compound rat mouse pellets (Special Diets Services Ltd., Essex, UK) and reverse osmosis filtered water. Intracranial injections were carried out using 8 to 10-week-old C57BL6/J mice, which were obtained from Charles River Laboratories (Wilmington, MA, USA) and were Jax purity assured. Mice were stereotactically injected with 1 × 10^5^ CT-2A cells in a volume of 2 µL into the right striatum (2.5 mm from the midline, 2.5 mm anterior from bregma, 3 mm deep). Surgery was performed under isoflurane general anaesthesia using aseptic techniques in accordance with the guiding principles of the Laboratory Animal Science Association document on aseptic surgery [22]. Animals were monitored daily for adverse effects, especially for signs of neurological symptoms and ill-health due to chronic intra-cranial pressure, such as reduced mobility, hunching, decrease in food intake, hypersensitivity upon touching and handling, separation from cage mates and significant weight loss. Analgesics were given peri-operatively as standard and post-operatively as required, determined by examination of animals for signs of distress. To limit suffering, animals were examined twice daily and adverse effects were scored according to the criteria in the license.

### 2.4. In Vivo Imaging Study Design

#### 2.4.1. MRI Acquisition

The MRI scans used for confirming the presence of an intra-cranial tumour were performed using a Bartec M7 Machine (1 Tesla, Bartec, Bad Mergentheim, Germany). For each animal, T2-weighted fast spin echo images obtained were acquired with the following parameters: 16 slices, 0.8 mm slice thickness, and 10 min acquisition time. MRI was acquired in 6 tumour-bearing mice, injected as previously described and multiple T1-weighted MRI acquisitions were performed to monitor tumour growth.

#### 2.4.2. Radiotracer Availability and PET-CT Acquisition

PET-CT imaging was performed on CT-2A tumour-bearing mice (*n* = 4), sham operated mice (*n* = 2), and healthy control mice (*n* = 1). ^18^F-fluciclovine PET tracer was provided by Blue Earth Diagnostics (Oxford, UK) through an agreement with University of Leeds. For pre-clinical administration, the radiotracer was reformulated by elution on chromatographic column to remove trisodium citrate to negate citrate poisoning in mice. The radiotracer was purified by elution on chromatographic column.

^18^F-fluciclovine (7.8 +/− 2.0 MBq, mean +/− standard deviation of injected activity) was intravenously injected (tail vein) at the beginning of a 90-min dynamic PET scan (Albira Si, Bruker), or 60 min before a 20-min static PET acquisition. All PET-CT acquisitions and injections were performed under 2% isoflurane anaesthesia at a flow rate of 2 L per minute. At the end of each PET acquisition, both dynamic and static, an extra 10-min Computed Tomography (CT) scan was performed. Dynamic and static PET scans were performed for evaluating ^18^F-fluciclovine biodistribution and uptake in healthy brains and tumours using an Albira SI PET/SPECT/CT scanner (Bruker, Ettlingen, Germany).

### 2.5. Immunohistochemistry

Representative CT-2A injected mouse brains were fixed with 4% paraformaldehyde (PFA) and taken through a dilution series of ethanol in preparation for paraffin embedding. These blocks were then processed in 5 μM sections and fixed onto glass slides. Immunohistochemistry staining followed the manufacturer’s instruction for the Mouse-on-Mouse Polymer IHC kit from Abcam (ab127055), including a 20′ incubation in Bloxall and a Casein blocking step. The following primary antibodies were used for ASCT2 (ab84903, concentration 1/100, Abcam, Cambridge, UK), Ki67 (ab16667, concentration 1/200, Abcam, Cambridge, UK) and Nestin (ab221660, concentration 1/2000, Abcam, Cambridge, UK). All primary antibodies were incubated overnight at 4 °C. Images were acquired on a Nikon TiE microscope (Nikon Corp, Tokyo, Japan) at ×10 magnification.

## 3. Results

### 3.1. Demographic and Oncological Outcomes

Table 1 describes the demographic and oncological data for the 6 patients. Mean age at diagnosis was 61 years (range 47–72 years) and 2/6 (33%) patients were female. All but one patient underwent surgical tumour debulking. The remaining patient (GBM 1), who had an eloquent tumour location, close to the primary motor cortex, underwent a biopsy alone. All patients attended every MRI visit however, 2 patients (GBM 1 and 2) did not have an end-of-treatment PET-CT owing to clinical deterioration. Two patients were still alive as of the censor date. Based on overall survival (OS), patients could be grouped into those with short survival (GBM 1–3, with a median OS of 249 days), and long survival (GBM 4–6, with a median OS of 903 days). Results from cytogenetic testing are outlined in Table 1.

All patients in the short survival group had unmethylated 6-O-methylguanine-DNA methyltransferase (MGMT) gene promoters and 2/3 in the long survival group had methylated MGMT promoters. MGMT methylation status testing failed in the third patient. All patients completed adjuvant chemoradiotherapy as planned. In addition, all 3 patients in the long survival group completed 6 cycles of adjuvant temozolomide while 2/3 patients in the short survival group did not receive adjuvant temozolomide due to clinical deterioration and the remaining 1/3 (GBM 3) received only 4 adjuvant cycles.

### 3.2. Clinical Imaging

#### 3.2.1. Radiologist Assessment

Table 2 outlines the summary of radiological assessment for MRI and PET-CT at each timepoint and also the volumes for Gd-T1, PET and DCE-MRI at each study timepoint.

**Figure 2:**
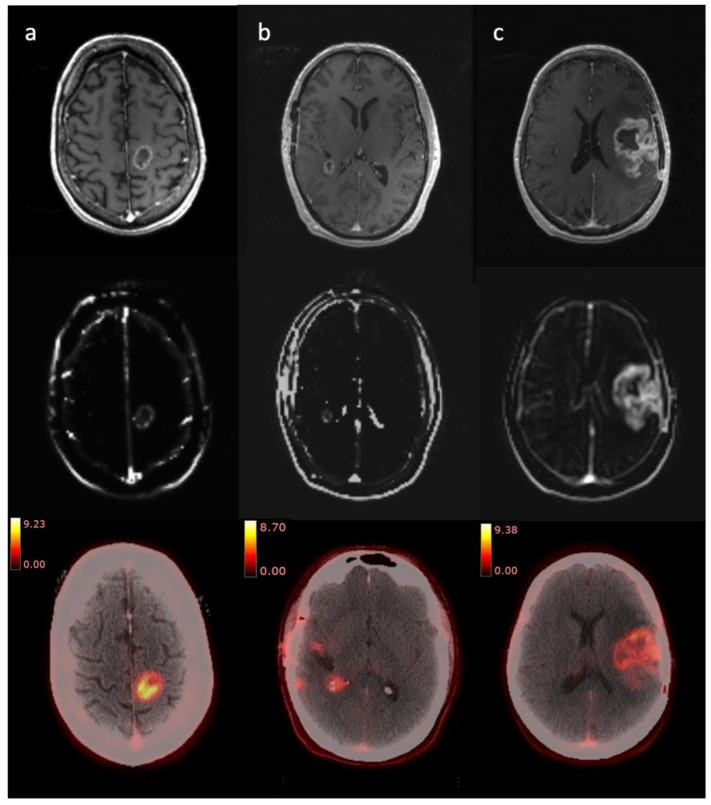
Selected Gd-T1 (top row), DCE-MRI (K^trans^, middle row) and PET-CT (bottom row, scale bar indicating SUV) images for three patients. (**a**) (left-hand column)—GBM 1, pre-radiotherapy; (**b**) (middle column)—GBM 3, pre-radiotherapy; (**c**) (right-hand column)—GBM 6, pre-radiotherapy.

In each of the short survival patients, the end-of-treatment studies were most consistent with disease progression based on radiologist interpretation. In the long survival group, one patient had stable disease (GBM 4), GBM 5 showed findings that were indeterminate between progression or pseudoprogression and GBM 6 had findings more consistent with progression on multiparametric MRI (mpMRI). For these latter two patients, PET-CT showed stable disease. Subsequent standard of care MRI for GBM 5 and 6 showed reduced tumour size. Figure 2 illustrates selected images from Gd-T1, DCE-MRI and PET-CT studies for selected patients.

#### 3.2.2. Tumour Volume Analysis

**Figure 3:**
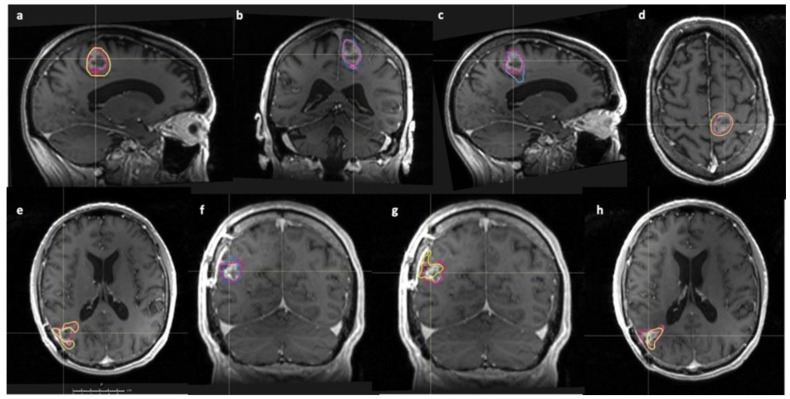
Selected Gd-T1 sagittal, axial and coronal images taken at different study timepoints, with PET or DCE-MRI volumes overlaid for GBM 1 (top row) and GBM 5 (bottom row). Yellow—PET (3 × SUV_max_) volume; Pink/red—Gd-T1 volume; Blue—K^trans^ volume. Top row—GBM 1; (**a**) –pre-RT sagittal, (**b**)—coronal pre-RT, (**c**)—sagittal mid-RT and (**d**)—axial mid-RT images. Bottom row—GBM 5; (**e**)—axial pre-RT, (**f**)—coronal mid-RT, (**g**)—coronal mid-RT and (**h**)—axial post-RT images.

When comparing short and long survival patients, the 3 × SUV_max_ PET volume showed the most consistent difference to DCE-MRI and Gd-T1 volumes across all timepoints. Figure 3 illustrates selected images with overlaid tumour volumes for GBM 1 and 5 at different timepoints.

##### Short Survival

For GBM 1–3 the PET volume (2 × SUV_max_) was consistently larger than either of the DCE-MRI volumes (K^trans^ or v_e_) and these were, in turn, larger than most T1-Gd volumes (*p* = 0.27). For GBM 1, the 3 × and 4 × SUV_max_ PET volumes were either larger than or nearly equal to the DCE-MRI volumes, and always larger than the Gd-T1 volume. For GBM 2, the 3 × SUV_max_ PET was predominantly larger than DCE-MRI, which were all, in turn, larger than Gd-T1. For GBM 3, the 3 × SUV_max_ volume was larger than v_e_ and Gd-T1. The 4 × SUV_max_ volume was slightly smaller or similar to most of the DCE-MRI and Gd-T1 volumes (K^trans^ post-RT was much larger).

##### Long Survival

For GBM 4–6, the 3 and 4 × SUV_max_ PET volumes were mostly smaller than the DCE-MRI volume and Gd-T1 volume for all timepoints (*p* = 0.79). The 2 × SUV_max_ volumes were also larger than all DCE-MRI and Gd-T1 volumes at most timepoints for GBM 5 and 6 (*p* = 0.89). The DCE-MRI and Gd-T1 volumes were similar in overall volume to each other. All volumes were noticeably smaller for GBM 4 than other patients and there were still areas of Gd uptake on Gd-T1 and DCE-MRI, even when the PET showed no uptake.

##### Subtracted Volumes

Gd-T1 volumes subtracted from both the DCE-MRI and PET volumes are described in Table 3.

**Figure 4:**
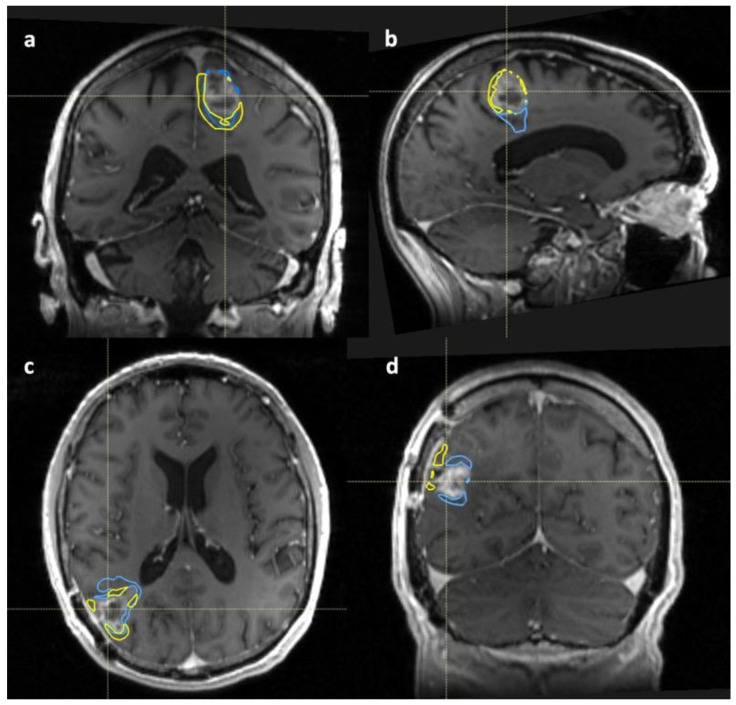
Selected Gd-T1 images from different study timepoints with subtracted DCE-MRI and PET volumes overlaid for GBM 1 (top row) and GBM 5 (bottom row). Volumes are subtracted from the Gd-T1 volume (not shown): Yellow—PET (3 × SUV_max_) volume; Blue—K^trans^ volume. Top row—GBM 1; (**a**)—pre-RT and (**b**)—mid-RT images. Bottom row—GBM 5; (**c**)—pre-RT and (**d**)—post-RT images.

When comparing the PET and each DCE-MRI volume outside of the enhancing tumour margin, the Dice similarity coefficient was consistently low for patients in both survival groups and across timepoints, suggesting unique areas of uptake. Figure 4 shows representative images from GBM 1 and GBM 5 with the subtracted DCE-MRI and PET (3 × SUV_max_) volumes overlaid on Gd-T1 images at different timepoints.

### 3.3. Pre-Clinical Results

**Figure 5:**
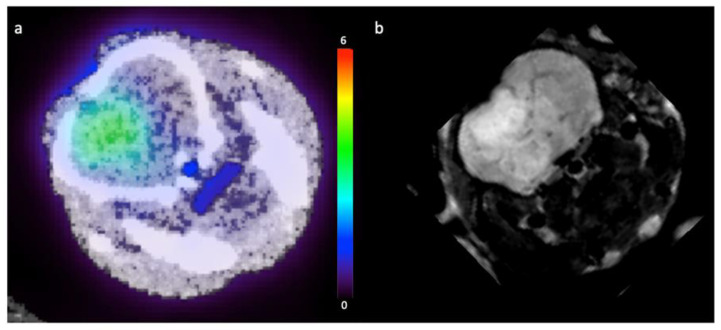
Selected images from the same CT-2A tumour-bearing mouse. (**a**)—Static ^18^F-fluciclovine PET-CT image (with standardised uptake value scale bar); (**b**)—and T2-weighted MRI from the same mouse.

Pre-clinical glioma model CT2A demonstrated increased ^18^F-fluciclovine tracer uptake within the tumour. Figure 5 shows a representative image taken from a static PET-CT reconstruction in a tumour-bearing mouse 60 min following injection of 9.3 MBq ^18^F-fluciclovine and a representative T2-weighted image from the same mouse. Similar appearances were demonstrated in the other tumour-bearing mice.

**Figure 6:**
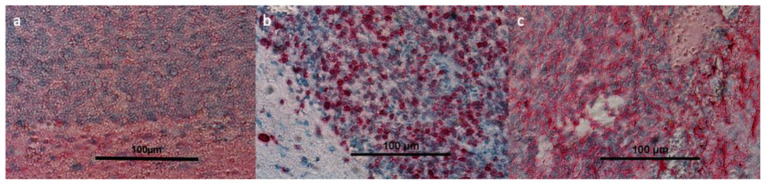
Slides demonstrating results of post-mortem immunohistochemical staining from tumours in a CT-2A pre-clinical mouse model. CT-2A mice brains were fixed with 4% paraformaldehyde (PFA), embedded in paraffin, and processed into 5 µM sections for Immunohistochemistry staining. Images were then taken on a Nikon TiE microscope at ×10 (scale bar = 100 µm). Representative images above cell nuclei in blue, cytoplasm in lighter blue and markers for ASCT2 (**a**), Ki-67 (**b**), nestin (**c**), in red.

Figure 6 shows representative immunohistochemistry staining for proliferation (Ki-67), stem cells (Nestin) and amino acid transporter (ASCT2) in post-mortem tumour taken from one of the tumour-bearing mice. These data confirm that uptake observed in this model was associated with relevant transporter protein expression and with areas of the proliferation of stem cell marker positive cells, and supports the view that uptake observed in the clinical cohort occurred in biologically active tumour volumes.

## 4. Discussion

Results from this pilot study suggest that ^18^F-fluciclovine uptake is not an epiphenomenon of BBB breakdown, even during and after adjuvant chemoradiotherapy. In the pre-clinical model, there was tumour-specific uptake which reflected tumour areas with high cellular proliferation and stem cell positivity, which also expressed relevant transporter proteins.

In the clinical cohort, tumours, defined by ^18^F-fluciclovine PET uptake, differed from tumours defined by MRI. There was tracer activity beyond the enhancing margin of GBM in the case of shorter OS patients (GBM 1–3). In these patients, the volume of ^18^F-fluciclovine PET uptake in relation to Gd-T1 and DCE-MRI tended to be larger, whereas the converse was observed for those with longer survival, and this relationship remained over the course of adjuvant chemoradiotherapy. This was dependent upon which PET threshold was used, and 3 × SUV_max_ appeared the most consistent volume threshold for which this pattern was observed.

It is important to state that patients in the short survival group all had unmethylated MGMT promoters, whereas they were methylated for GBM 4 and 5, which will have contributed to the survival difference between groups. The MGMT gene encodes proteins, which remove alkyl groups from guanine and are therefore integral to the DNA repair process. The silencing of the MGMT gene via promoter methylation limits this repair process and allows greater DNA damage by alkylating chemotherapeutic agents and confers a better prognosis [23]. Hence the different survival times between the two groups in our study are likely to be explained by this. However, the MGMT statuses of the groups do not detract from the observed differences in the imaging findings.

The finding of larger PET-derived tumour volumes in the context of decreased OS, appears consistent with other studies that compared ^18^F-fluciclovine uptake to areas of Gd-T1 enhancement [4,24]. Kondo et al. reported that the extent of ^18^F-fluciclovine uptake using a 10-min post-injection reconstruction was generally larger than for Gd-T1 for patients with GBM and anaplastic astrocytomas [4]. Additionally, there were 3 cases of anaplastic astrocytoma that had histologically confirmed tumour tissue in regions with ^18^F-fluciclovine uptake but no enhancement on Gd-T1 MRI. Of 28 samples taken within ^18^F-fluciclovine uptake, 27 were positive for tumour cells (96.4% positive predictive value). Wakabayashi et al. investigated ^18^F-fluciclovine and Gd-T1 enhancement in the context of predominantly grade 2 and 3 astrocytoma or oligodendroglioma, with some cases of GBM, and demonstrated a 100% positive predictive value upon histology for areas of ^18^F-fluciclovine uptake that showed no enhancement on the Gd-T1 sequence [24]. 

The aforementioned studies were performed on pre-operative images, and other groups have shown ^18^F-fluciclovine uptake in recurrent GBM, however, studies examining the activity of ^18^F-fluciclovine during adjuvant chemoradiotherapy are limited [8,25,26]. Nabavizadeh et al. performed 60-min dynamic ^18^F-fluciclovine PET-CT in patients with new enhancing lesions on Gd-T1 MRI following completion of adjuvant radiotherapy and who subsequently underwent resection [26]. They reported a positive correlation between SUV_peak_ and percentage of tumour in resected specimens, and also a significantly higher SUV_peak_ in patients with true progression (tumour represented ≥50% of the specimen) compared to those with pseudoprogression (tumour represented ≤10% of the specimen). Other groups have also shown ^18^F-fluciclovine uptake in recurrent GBM following adjuvant treatment, outside of areas of Gd-T1 hyperintensity [9].

These studies suggest that ^18^F-fluciclovine uptake outside of Gd-T1 enhancement largely correlates with glioma tissue, a finding supported by our pre-clinical glioma model. Larger PET volumes in the short survival cohort suggest that there is a greater volume of active tumour in these patients than that shown with Gd-T1. The pre-clinical glioma model also showed ^18^F-fluciclovine uptake in a biologically active tumour, with positive staining for markers of proliferation, stem cells and the ASCT2 uptake protein. In other animal glioma models, ^18^F-fluciclovine has been shown to correlate with tumour cell density rather than markers of BBB breakdown, implying that different components of a glioma may be highlighted using ^18^F-fluciclovine compared to Gd-T1 [11]. Conversely, the opposite may be the case in the longer OS patients, in which Gd-T1 volumes tended to be larger, and there may have been fewer glioma tumour cells present than MRI suggested. Further study is necessary to corroborate these findings, particularly with histological confirmation during adjuvant therapy, to determine whether ^18^F-fluciclovine can more accurately delineate radiotherapy treatment volumes or could be used to monitor treatment response or progression during adjuvant treatment [27]. Obtaining additional tissue samples beyond routine surgical care presents an ethical challenge, however, and as shown in this work, pre-clinical studies may offer an alternative experimental avenue.

The dynamic PET acquisition and time-activity curves (Figure 1) were in concordance with reported literature, with a stable equilibrium of activity achieved 5-min after ^18^F-fluciclovine injection and a 10-min static reconstruction used for image interpretation [3]. Other groups have used a later timepoint for ^18^F-fluciclovine PET acquisition, however, this would not have changed the overall activity based on our time-activity curves. For volume delineation, our PET threshold was based on the ratio of the SUV_max_ of background tissue, whereas other groups have used SUV_mean_ of background tissue [9]. This difference can be explained by the use of an earlier, 10-min static reconstruction for image interpretation. The use of background SUV_mean_ would have resulted in spuriously large tumour volumes that would not have corresponded to anatomical boundaries.

An exploratory aim of this study was to assess whether any areas outside of the region of BBB breakdown (as marked by Gd-T1 hyperintensity) showed any strong concordance between ^18^F-fluciclovine and DCE-MRI parameter maps. The relationship between Gd-T1, DCE-MRI and ^18^F-fluciclovine PET-CT during adjuvant chemoradiotherapy for GBM has not previously been reported to the best of our knowledge, and comparison of the PET and DCE-MRI volumes outside of the Gd-T1 volume demonstrated low (0–0.5) Dice similarity coefficient (Table 3). The results suggest that there is poor concordance between the areas outside of the Gd-T1 enhancing margins delineated by ^18^F-fluciclovine PET and DCE-MRI sequences, although the differences in resolution across the modalities may contribute to findings. High-grade gliomas (such as GBM) are characterised by disorganised vasculature, immature endothelial cells and increased permeability, with the margins associated with increased microvessel density (MVD) and neovascularization [28]. Studies of the tissue surrounding the enhancing portion of GBM have shown correlations between DCE-MRI parameters and expression of hypoxia-related proteins such as vascular endothelial growth factor-A (VEGF-A), suggesting that the DCE-MRI changes could be detecting regions of tissue hypoxia [12,13,29]. The finding of discordant DCE-MRI and ^18^F-fluciclovine PET volumes outside of the Gd-T1 hyperintense regions raises the question of whether the two approaches could be used in a complementary fashion, potentially highlighting regions of increased glioma cellular density and areas of increased tissue hypoxia and therefore highlight different tumour niches. Indeed, different tumour niches have been described for GBM including the perinecrotic niche and the perivascular tumour niche, emphasising the intratumoural heterogeneity that exists in GBM [30].

Other advanced MRI sequences such as amide proton transfer weighted (APTw) imaging were not used in our study but have demonstrated promising results in the non-invasive characterisation of diffuse glioma and GBM [31,32]. APTw is a specific type of chemical exchange saturation transfer (CEST) MRI sequence that produces a signal based on the amount of endogenous mobile protein and peptides within tissues, and the signal has been correlated with tumour cell density and proliferation in gliomas [33]. High APTw signal has been correlated with increased tumour cellularity in non-enhancing parts of high-grade gliomas, and therefore it may be possible to obtain an image of the extent of tumour infiltration [32].

Our findings also suggest a potential correlation between the different survival groups and DCE-MRI volumes during adjuvant chemoradiotherapy treatment. As expected in the short survival group, the DCE-MRI and Gd-T1 volumes increased in volume throughout treatment, whereas these were reduced for long survival patients. This is in keeping with other studies of DCE-MRI volumes during adjuvant treatment for GBM. For example, Kim et al. monitored changes in the hyperperfused and hypercellular tumour volume, which were identified using a combination of DCE-MRI and diffusion-weighted imaging (DWI). Patients with a greater reduction in the hyperperfused and hypercellular volume demonstrated better OS [16]. Others have used specific DCE-MRI metrics such as K^trans^ and v_e_ and changes in these over the course of treatment, rather than changes in volume, to stratify patients based on response and survival [14,15]. Yoo et al. found a significantly lower mean v_e_ value in enhancing tissue following completion of concurrent chemoradiotherapy and adjuvant temozolomide in patients with progression during the follow-up period [34]. In our study, the DCE-MRI and Gd-T1 volume changes showed similar trends in the short and long survival groups, however, it is known that Gd-T1 changes during treatment can be misleading and may increase due to pseudo-progression or radionecrosis [35]. Using DCE-MRI in conjunction with Gd-T1 may provide an adjunctive sequence to determine response or progression during treatment. As shown in Table 2, the radiologist’s interpretation of the MRI at the time of acquisition, which did not consider the DCE-MRI sequences, could not always distinguish disease progression and pseudoprogression.

This study has several limitations. GBM is a heterogenous condition that varies both within an individual tumour (intratumoral heterogeneity) and between patients (intertumoral heterogeneity). Hence, since only six patients were recruited, it is difficult to make generalisations from such a small sample; this pilot study was undertaken as a hypothesis-generating study. The observed trends were small, and a group-wise statistical difference was not observed, however it must be noted that the study was not powered to find statistically significant differences in imaging volumes. It has been possible to demonstrate the feasibility of imaging patients with ^18^F-fluciclovine PET-CT and mpMRI before, during and after adjuvant chemotherapy. Histological confirmation was not obtained from study participants of the fluciclovine uptake or DCE-MRI changes which limits the ability to determine any pathological correlation to imaging findings. However, this reflects routine clinical practice during adjuvant treatment. ^18^F-fluciclovine PET-CT was not acquired pre-operatively to allow comparison of non-treated tumour volume and the changes observed during treatment. However, it is routine practice to use the post-operative, pre-adjuvant chemoradiotherapy study as a new baseline for assessing response to treatment and therefore our study is useful in this context. Due to differences in delineation between PET (using semi-automatic thresholding) and the Gd-T1 and DCE-MRI (manual segmentation), there is the possibility of PET uptake being included from regions that do not correspond to the brain parenchyma, such as the surgical resection cavity or ventricle, which would be omitted by the MRI volumes. However, the use of a semi-automatic threshold for PET contouring reduces the chance of any interobserver error influencing the results of volume comparison for PET volumes. There are also inherent differences in the imaging resolution of the modalities that we have compared (PET, DCE-MRI, T1-Gd) and it is possible that some of our findings in the comparison of contours are due to differing resolution. Additionally, as the software used to process DCE-MRI (PMI) could only generate JPEG images, our ability to adjust window width and level may have been affected and this could have impacted our ability to manually contour the DCE-MRI images. The ability to delineate areas of uptake on DCE-MRI may have been hampered by the loss of image data in the conversion from JPEG to DICOM file format. The study only included patients with focal GBM, and future studies should include cases of multifocal GBM or unenhancing distant disease to assess the ^18^F-fluciclovine uptake at these sites also. Lastly, the interval of follow-up with the last ^18^F-fluciclovine and mpMRI following adjuvant treatment was relatively short. This interval of imaging surveillance could be extended in future assessments.

## 5. Conclusions

In conclusion, in this exploratory analysis, ^18^F-fluciclovine appeared to show uptake beyond Gd-T1 before, during and after adjuvant chemoradiotherapy treatment, in a manner that was dependent on the prognosis of the patient. Short survival patients tended to show an increased volume of ^18^F-fluciclovine compared with mpMRI, whereas the converse was seen in long survival patients. This finding was most consistent when using a PET threshold of 3 × SUV_max_ of contralateral normal brain to threshold PET uptake. In areas outside of the Gd-T1 hyperintensity, there was poor concordance between the DCE-MRI and ^18^F-fluciclovine volumes. Future studies will be needed to validate ^18^F-fluciclovine activity, particularly with immunohistochemical correlation as these imaging modalities may provide more accurate tumour definition as therapies move towards more individualisation.

## Figures and Tables

**Table 1:** Demographic and oncological data.

	Patient					
	GBM 1	GBM 2	GBM 3	GBM 4	GBM 5	GBM 6
Age at surgery (years)	72	47	54	67	57	68
Gender	M	M	M	F	M	F
PET-CT studies (number)	2	2	3	3	3	3
Multiparametric MRI studies (number)	3	3	3	3	3	3
Status	Deceased	Deceased	Deceased	Alive	Alive	Deceased
Overall survival (days)	249	193	292	910 *	903 *	554
Surgery	Stereotactic biopsy	Resection	Resection	Resection	Resection	Resection
Radiotherapy	60Gy/30 fractions	60Gy/30 fractions	60Gy/30 fractions	60Gy/30 fractions	60Gy/30 fractions	60Gy/30 fractions
Adjuvant temozolomide	None	None	4 cycles	6 cycles	6 cycles	6 cycles
Histology	Glioblastoma	Glioblastoma	Glioblastoma	Glioblastoma	Glioblastoma	Glioblastoma
Cytogenetic analysis						
IDH1/2	Wild type	Wild type	Wild type	Wild type	Wild type	Failed
MGMT	Unmethylated	Unmethylated	Unmethylated	Methylated	Methylated	Failed
TERT promoter	Mutated	Mutated	Mutated	Mutated	Wild type	Failed
1p/19q co-deletion	Wild-type	Wild-type	Wild-type	Wild-type	Wild-type	Failed

* Survival time calculated up to 14 June 2021. IDH—isocitrate dehydrogenase. MGMT—6-O-methylguanine-DNA methyltransferase. TERT—Telomerase reverse transcriptase. Gy—Gray.

**Table 2:** Tumour volumes for PET and MRI for each timepoint (cm^3^).

	**GBM 1**							
**PET-CT**					**MRI**			
**Timepoint**	**2 × SUV_max_**	**3 × SUV_max_**	**4 × SUV_max_**	**Radiologist Assessment**	**Gd-T1**	**K^trans^**	**v_e_**	**Radiologist Assessment**
Pre-RT	16.0	11.1	8.9	Avid tumour	6.3	7.3	7.8	Stable
Mid-RT	14.9	10.6	7.0	Stable	6.8	7.1	5.6	Stable
Post-RT	-	-	-	-	13.5	14.1	15.5	Progression
	**GBM 2**							
**PET-CT**					**MRI**			
**Timepoint**	**2 × SUV_max_**	**3 × SUV_max_**	**4 × SUV_max_**	**Radiologist Assessment**	**Gd-T1**	**K^trans^**	**v_e_**	**Radiologist Assessment**
Pre-RT	47.7	36.2	29.0	Avid tumour	21.4	23.3	25.8	Progression
Mid-RT	71.8	57.4	44.5	Progression	47.9	53.1	57.4	Progression/Pseudoprogression
Post-RT	-	-	-	-	84.3	84.8	96.9	Progression
	**GBM 3**							
	**PET-CT**				**MRI**			
**Timepoint**	**2 × SUV_max_**	**3 × SUV_max_**	**4 × SUV_max_**	**Radiologist Assessment**	**Gd-T1**	**K^trans^**	**v_e_**	**Radiologist Assessment**
Pre-RT	12.1	6.1	3.4	Multifocal avid tumour	3.4	2.8	3.9	Mixed picture
Mid-RT	13.0	6.5	3.6	Stable	4.9	5.1	4.5	Progression
Post-RT	31.0	18.4	12.8	Progression	13.4	23.3	12.5	Progression
	**GBM 4**							
	**PET-CT**				**MRI**			
**Timepoint**	**2 × SUV_max_**	**3 × SUV_max_**	**4 × SUV_max_**	**Radiologist Assessment**	**Gd-T1**	**K^trans^**	**v_e_**	**Radiologist Assessment**
Pre-RT	0.6	0.1	0	Likely remnant tumour	0.4	0.6	0.8	Small volume enhancement
Mid-RT	0.2	0	0	Stable	0.1	0.2	0.1	Stable
Post-RT	0.1	0	0	Stable	0.1	0.1	0.03	Stable
	**GBM 5**							
	**PET-CT**				**MRI**			
**Timepoint**	**2 × SUV_max_**	**3 × SUV_max_**	**4 × SUV_max_**	**Radiologist Assessment**	**Gd-T1**	**K^trans^**	**v_e_**	**Radiologist Assessment**
Pre-RT	12.4	3.2	0.5	Uptake at margins	6.3	11.6	9.9	Stable
Mid-RT	12.8	5.1	1.4	Stable	5.1	4.0	5.3	Stable
Post-RT	7.2	1.8	0.2	Stable	5.1	4.6	5.5	Progression/pseudoprogression
	**GBM 6**							
	**PET-CT**				**MRI**			
**Timepoint**	**2 × SUV_max_**	**3 × SUV_max_**	**4 × SUV_max_**	**Radiologist Assessment**	**Gd-T1**	**K^trans^**	**v_e_**	**Radiologist Assessment**
Pre-RT	44.4	35.0	27.5	Large avid tumour	46.6	51.0	48.3	Stable/Large residuum
Mid-RT	43.9	33.7	26.3	Partial response	32.9	36.6	34.6	Stable
Post-RT	46.8	36.8	28.6	Stable tumour	35.4	40.0	39.3	Likely progression

SUV_max_—maximum standard uptake value. Gd-T1—T1-weighted post-gadolinium sequence. K^trans^—volume transfer constant. v_e_—interstitial volume per unit volume of tissue. Radiologist assessment did not make use of DCE-MRI sequences.

**Table 3:** Volumes of PET and DCE-MRI tumour volume outside of the Gd-T1 volume, and their similarity.

	**GBM 1**				
	**Volume** (**cm^3^**)			**Dice Similarity Coefficient** (**DSC**)	
**Timepoint**	**PET** (**3 × SUV_max_**)	**K^trans^**	**v_e_**	**PET vs. K^trans^**	**PET vs. v_e_**
Pre-RT	5.1	1.4	1.6	0.4	0.3
Mid-RT	4.1	2.1	1.1	0.2	0.2
Post-RT	-	1.9	2.6	-	-
	**GBM 2**				
	**Volume** (**cm^3^**)			**Dice Similarity Coefficient** (**DSC**)	
**Timepoint**	**PET** (**3 × SUV_max_**)	**K^trans^**	**v_e_**	**PET vs. K^trans^**	**PET vs. v_e_**
Pre-RT	15.7	5.4	6.6	0.5	0.5
Mid-RT	12.0	9.5	12.6	0.5	0.5
Post-RT	-	7.3	16.1	-	-
	**GBM 3**				
	**Volume** (**cm^3^**)			**Dice Similarity Coefficient** (**DSC**)	
**Timepoint**	**PET** (**3 × SUV_max_**)	**K^trans^**	**v_e_**	**PET vs. K^trans^**	**PET vs. v_e_**
Pre-RT	3.8	1.0	2.3	0.2	0.3
Mid-RT	2.9	2.3	1.3	0.3	0.3
Post-RT	1.0	10.4	3.9	0.1	0.1
	**GBM 4**				
	**Volume** (**cm^3^**)			**Dice Similarity Coefficient** (**DSC**)	
**Timepoint**	**PET** (**3 × SUV_max_**)	**K^trans^**	**v_e_**	**PET vs. K^trans^**	**PET vs. v_e_**
Pre-RT	0.1	0.4	0.5	0	0
Mid-RT	-	0.1	0.04	-	-
Post-RT	-	0.04	0.03	-	-
	**GBM 5**				
	**Volume** (**cm^3^**)			**Dice Similarity Coefficient** (**DSC**)	
**Timepoint**	**PET** (**3 × SUV_max_**)	**K^trans^**	**v_e_**	**PET vs. K^trans^**	**PET vs. v_e_**
Pre-RT	1.9	6.0	5.0	0.2	0.2
Mid-RT	2.3	0.7	1.4	0.2	0.3
Post-RT	2.3	1.2	1.8	0.1	0.1
	**GBM 6**				
	**Volume** (**cm^3^**)			**Dice Similarity Coefficient** (**DSC**)	
**Timepoint**	**PET** (**3 × SUV_max_**)	**K^trans^**	**v_e_**	**PET vs. K^trans^**	**PET vs. v_e_**
Pre-RT	0.7	7.6	7.1	0.1	0.1
Mid-RT	4.4	7.3	6.3	0.5	0.5
Post-RT	4.9	7.1	6.1	0.3	0.4

SUV_max_—maximum standard uptake value. K^trans^—volume transfer constant. v_e_—interstitial volume per unit volume of tissue.

## Data Availability

The data presented in this study are available on request from the corresponding author. The data are not publicly available due to privacy restrictions.
